# Adjustments to the preanalytical phase of quantitative cell-free DNA analysis

**DOI:** 10.1016/j.dib.2015.12.009

**Published:** 2015-12-17

**Authors:** Abel Jacobus Bronkhorst, Janine Aucamp, Piet J. Pretorius

**Affiliations:** Centre for Human Metabolomics, Biochemistry Division, North-West University, Potchefstroom 2520, South Africa

## Abstract

Evaluating the kinetics of cell-free DNA (cfDNA) in the blood of cancer patients could be a strong auxiliary component to the molecular characterization of cfDNA, but its potential clinical significance is obscured by the absence of an analytical consensus. To utilize quantitative cfDNA assessment with confidence, it is crucial that the preanalytical phase is standardized. In a previous publication, several preanalytical variables that may affect quantitative measurements of cfDNA were identified, and the most confounding variables were assessed further using the growth medium of cultured cancer cells as a source of cfDNA (“Cell-free DNA: Preanalytical variables” [1]). The data accompanying this report relates to these experiments, which includes numerous changes to the sample handling and isolation protocols, and can be used for the interpretation of these results and other similar experiments by different researchers.

## Specifications table

**Table:** 

Subject area	Biochemistry, molecular biology
More specific subject area	Clinical biochemistry, translational oncology, prenatal diagnostics
Type of data	Excel spreadsheet, table
How data was acquired	PCR amplification of cell-free DNA was measured using a real-time quantitative assay for the β-globin gene. All assays were performed on a Rotor-Gene Q detection system (Qiagen) using a 72 well ring-setup.
Data format	Analyzed
Experimental factors	Centrifugation, medium storage temperature, medium thawing temperature, medium storage tube type, treatment with denaturing agents, combining snap freezing with proteinase K, binding buffer type, elution volume, elution regime and elution tube type.
Experimental features	Cell-free DNA was extracted directly from growth medium collected from 143B osteosarcoma cells in culture, and then quantified by real-time PCR. Several variations to the standard procedure were evaluated.
Data source location	South Africa
Data accessibility	The data is with this article

## Value of the data

• This data will be useful considerations when optimizing protocols and setting up a standard operating procedure, which should expedite the translation of cfDNA analyses to clinical practice.
• This data could be compared to other studies that investigated the effect of methodological variables on quantitative measurements of cfDNA.
• This data could be used to interpret studies that investigated the effect of methodological variables on qualitative measurements of cfDNA.

## 1 Data

In order to investigate the effects of several adjustments to the preanalytical phase of quantitative cfDNA measurements, the growth medium of cultured cancer cells was used as a source of cfDNA. The data in this report was obtained by amplifying cfDNA with real-time PCR, after it had been extracted under different preanalytical conditions. The data is presented in a supplementary file as a single table, which includes several quantitative measurements of cfDNA following modifications to the standard protocol followed. These changes are described in Table 1.

## 2 Experimental design, materials and methods

### 2.1 Cell culturing

Culture medium of the human bone cancer (osteosarcoma) cell line 143B (ATCC® CRL-8303™) was used as a source of cfDNA. Given that DNA levels in growth medium fluctuate much like cfDNA in the blood of humans, we could use it as a model to evaluate the effect of different variables on both high and low concentrations of cfDNA. Cells were cultured in T75 flasks in Dulbecco׳s Modified Eagle׳s medium (DMEM) (HyClone; #SH30243.01) supplemented with 10% fetal bovine serum (Biochrom; #S0615) and 1% penicillin/streptomycin (Lonza; #DE17-602E) at 37 °C in humidified air and 5% CO_2_. After the cells have reached the necessary confluency, the culture medium was removed, processed and stored at −80 °C in 15 ml tubes.

### 2.2 Extraction of cfDNA

CfDNA was extracted with the NucleoSpin Gel and PCR Clean-up kit (Macherey-Nagel, Düren, Germany; #1502/001), according to the instructions described by the PCR clean-up user manual. Briefly, samples were removed from the −80 °C freezer and thawed at 37 °C and then vortexed and centrifuged briefly. For each biological replicate, cfDNA was extracted in triplicate. For every sample, 600 µL of growth medium was mixed with 1200 µL of binding buffer. Samples were then vortexed, the entire volume of growth media was added to the spin column in small regiments, and centrifuged at 11 000×g for 1 min at room temperature. The columns were then washed twice, followed by the elution of cfDNA into 20 µL of elution buffer.

### 2.3 Quantification of cell-free DNA

PCR amplification of cfDNA was measured using a real-time quantitative assay for the β-globin gene. All assays were performed on a Rotor-Gene Q detection system (Qiagen) using a 72 well ring-setup. The reaction mixture consisted of 2 μL DNA and 23 μL master mix, which was composed of 8.1 μL H2O, 12.5 μL TaqMan Universal MasterMix (Life technologies; #1502032), 0.4 μL of 10 μM dual fluorescent probe 5′-(FAM)AAG GTG AAC GTG GAT GAA GTT GGT GG(TAMRA)-3′, and 1 μL of 10 μM forward and reverse primers, respectively. The primers used were: F1, 5′-GTG CAC CTG ACT CCT GAG GAG A-3′, and R1, 5′-CCT TGA TAC CAA CCT GCC CAG-3′. These probe and primers were synthesized by Integrated DNA Technologies (IDT, Whitehead Scientific). PCR conditions were set to: 95 °C for 10 min, followed by 45 cycles of 15 s denaturation at 95 °C, 1 min annealing at 60 °C, followed by 30 s extension at 72 °C. Sequence data of β-globin is attainable from GenBank (accession number: U01317). The absolute concentration of the target gene was calculated by using a standard curve. In this study, a standard curve was generated using five-fold serial dilutions of genomic DNA (50,000, 5000, 500, 50 and 5 pg/µL). Each biological replicate was quantified in duplicate, and triplicates of the standard curve were included in each run (only assays with *R*^2^ values>0.99 for the standard curve were used).

## Figures and Tables

**Table 1:** Modifications to standard procedure.

**Modifications to sample handling**	
**Modification to standard procedure**	**Description**
Centrifugation regime	Growth medium was centrifuged for 10 min at different forces (1000, 5000, 10 000 and 20,000×g). Other samples were subject to two rounds of centrifugation, first at 1000×g and then transferred to new tubes before the next centrifugation at 5000, 10,000 and 20,000×g, respectively. After centrifugation all samples were transferred to new tubes.
Growth medium storage temperature	After centrifugation, growth medium was transferred to fresh tubes and stored until cfDNA was extracted. Three storage schemes were tested: −20 °C, −80 °C, and snap-freezing in liquid nitrogen followed by storage at −80 °C.
Growth medium thawing temperature	Prior to cfDNA extraction, the growth medium is thawed. Two approaches were tested: thawing of growth medium at room temperature, and at 37 °C in a temperature controlled water bath for 5 min.
Growth medium storage tube type	After collection and processing, growth medium was stored in three different tubes: 15 mL nuclease free tubes (Ambion), regular 1.5 mL tubes (Eppendorf), and DNA LoBind tubes (Eppendorf).
	
**Modifications to cfDNA extraction protocol**	
Treatment with denaturing agents	Prior to cfDNA extraction, growth medium was treated with SDS (0.05%), proteinase K (1.5 mg/mL), and a combination of the two for 30 min at 50 °C, respectively. In the cases where SDS was used, buffer NTB was used instead of buffer NTI. As the kit makes no suggestions regarding the use of proteinase K, buffer NTI was used in this case.
Effect of combining snap freezing with proteinase K	Four different scenarios were compared: (1) cfDNA was extracted from growth medium directly after collection, (2) Growth medium was treated with proteinase K immediately after collection, followed by cfDNA extraction, (3) Growth medium was snap frozen before cfDNA extraction, and (4) Growth medium was snap frozen and then thawed and treated with proteinase K prior to extraction.
Binding buffer type	After thawing, growth medium is mixed with binding buffer before it is added to the spin column. Here, we compared two binding buffers, NTI and NTB. In the case of buffer NTB, the ratio of sample to buffer is 1:5. In the case of extractions where buffer NTI is used, the sample to buffer ratio is only 1:2.
Elution volume	CfDNA was extracted and eluted into 20 μL, 40 μL, 60 μL, and 100 μL of elution buffer, respectively.
Elution regime	CfDNA was extracted and eluted into 20 μL of elution buffer and repeated twice more to have a final volume of 60 μL. This was followed by the elution of DNA into 30 μL elution buffer and repeated once more to achieve a final volume of 60 μL. The former was compared to DNA eluted into 60 μL of elution buffer once.
Elution tube type	To examine the loss of eluted DNA due to adsorption to tube walls, regular 1.5 mL tubes (Eppendorf) were compared with 1.5 mL DNA LoBind tubes (Eppendorf)
	
**Comparing different protocols**	
Non-optimized	Media was collected and centrifuged at 1000×g and transferred to fresh 1.5 mL Eppendorf DNA LoBind tubes and stored at −20 °C. Before extraction, the medium was thawed at room temperature, and no denaturing agent was added thereafter. CfDNA was then extracted and eluted into 20 μL of elution buffer in one step. Samples were stored in 1.5 mL DNA LoBind tubes (Eppendorf)
Optimized	Media was collected and centrifuged at 10,000×g and transferred to fresh 15 mL tubes (Ambion). The media was then snap-frozen in liquid nitrogen and stored at −80 °C. The samples were then thawed at 37 °C, and incubated with proteinase K (1.5 mg/mL) for 30 min at 37 °C. CfDNA was extracted and eluted into 60 µL of elution buffer in three steps (3×20 µL) into regular 1.5 mL tubes (Eppendorf)
QIAmp DSP virus kit	CfDNA was extracted according to the instructions provided by the manufacturer.
	
**Increasing the yield of cfDNA**	
Effect of media evaporation	For each replicate, 6 mL of growth medium was aliquot into 2 mL tubes and evaporated in a SpeedVac to a total volume of 2.5 mL
